# Proximal and distal regulation of the HYAL1 gene cluster by the estrogen receptor α in breast cancer cells

**DOI:** 10.18632/oncotarget.12630

**Published:** 2016-10-13

**Authors:** Lydia Edjekouane, Samira Benhadjeba, Maïka Jangal, Hubert Fleury, Nicolas Gévry, Euridice Carmona, André Tremblay

**Affiliations:** ^1^ Research Center, CHU Sainte-Justine, Montréal, Québec, H3T 1C5 Canada; ^2^ Department of Biochemistry and Molecular Medicine, Faculty of Medicine, University of Montreal, Montréal, Québec, H3T 1J4 Canada; ^3^ Department of Biology, Faculty of Sciences, University of Sherbrooke, Sherbrooke, Québec, J1K 2R1 Canada; ^4^ CHUM Research Center, Institut du cancer de Montréal, Montréal, Québec, H2X 0A9 Canada; ^5^ Department of Obstetrics & Gynecology, Faculty of Medicine, University of Montreal, Montréal, Québec, H3T 1J4 Canada

**Keywords:** hyaluronidase, estrogen receptors, 3p21.3 cluster

## Abstract

Chromosomal and genome abnormalities at the 3p21.3 locus are frequent events linked to epithelial cancers, including ovarian and breast cancers. Genes encoded in the 3p21.3 cluster include HYAL1, HYAL2 and HYAL3 members of hyaluronidases involved in the breakdown of hyaluronan, an abundant component of the vertebrate extracellular matrix. However, the transcriptional regulation of HYAL genes is poorly defined. Here, we identified the estrogen receptor ERα as a negative regulator of HYAL1 expression in breast cancer cells. Integrative data mining using METABRIC dataset revealed a significant inverse correlation between ERα and HYAL1 gene expression in human breast tumors. ChIP-Seq analysis identified several ERα binding sites within the 3p21.3 locus, supporting the role of estrogen as an upstream signal that diversely regulates the expression of 3p21.3 genes at both proximal and distal locations. Of these, HYAL1 was repressed by estrogen through ERα binding to a consensus estrogen response element (ERE) located in the proximal promoter of HYAL1 and flanked by an Sp1 binding site, required to achieve optimal estrogen repression. The repressive chromatin mark H3K27me3 was increased at the proximal HYAL1 ERE but not at other EREs contained in the cluster, providing a mechanism to selectively downregulate HYAL1. The HYAL1 repression was also specific to ERα and not to ERβ, whose expression did not correlate with HYAL1 in human breast tumors. This study identifies HYAL1 as an ERα target gene and provides a functional framework for the direct effect of estrogen on 3p21.3 genes in breast cancer cells.

## INTRODUCTION

Mammalian hyaluronidases are involved in the hydrolysis of the glycosaminoglycan hyaluronan, a critical component of the extracellular matrix that regulates cell growth, migration, and differentiation, and other processes such as extracellular water and protein homeostasis, cartilage and vascular integrity. Family members include HYAL1, HYAL2, and HYAL3, clustered in the 3p21.3 chromosomal region, and HYAL4, SPAM1 (sperm adhesion molecule 1; also known as PH-20/HYAL5) and HYALP1, encoded on chromosome 7q31.3. Of these, only HYAL1, HYAL2 and SPAM1 act as *bona fide* hyaluronidases with genuine endo-N-acetylhexosaminidase activity, whereas HYAL3 is considered inactive, HYAL4 possesses chondroitinase activity and HYALP1 is a pseudogene [1, 2]. HYAL1 acts as the most potent hyaluronidase, being highly present in a broad range of tissues and in plasma, and exhibiting wider substrate recognition, which suggests a central role of HYAL1 in hyaluronan fragmentation and extracellular matrix turnover [3, 4]. Mutations in human hyaluronidase-coding genes have as yet been identified only in HYAL1, resulting in lysosomal disorders and juvenile idiopathic arthritis [5, 6].

Increasing evidence supports a role of hyaluronidases in tumorigenesis and metastatic potential mostly associated with changes in hyaluronan breakdown profile. Intriguingly, expression levels of hyaluronidases are variable in a cancer type-dependent fashion, providing them with either oncogenic or tumor suppressor activity. Increased HYAL1 levels were found to correlate with tumor aggressiveness and poor survival in head and neck, prostate and bladder cancer [7–9], whereas HYAL1 expression was decreased in advanced ovarian carcinomas and in endometrial cancer [10–12]. Chromosomal aberrations and instability at the 3p21.3 locus and homozygous deletions targeting HYAL1/2/3 have been frequently found in many epithelial cancers, suggesting a potential role of tumor suppressor for the genes encoded at this locus [13–15]. In ovarian cancer, allelic imbalance of the HYAL1/2/3 clustered genes was reported in tumor and stroma tissues, and in particular, HYAL1 expression was significantly reduced in serous epithelial ovarian cancer compared to normal ovaries or to other ovarian cancer subtypes [10, 16, 17]. Consistent with such HYAL1 reduction, extracellular accumulation of hyaluronan is often observed in ovarian tumor stroma and pericellular matrix with correlation to poor disease outcome [3, 18].

Aberrant expression of HYAL1, HYAL2 and SPAM1 has also been reported in breast cancer, and in particular upregulation of HYAL1 was observed in infiltrating invasive duct cancer tissues and metastatic lymph nodes [19, 20]. Overexpression of HYAL1 also induced migration of breast cancer cells and promoted xenograft tumor size and angiogenesis [21]. Therefore, the effect of HYAL1 appears to be highly context-dependent in terms of cancer type and progression. Although aberrant HYAL1 expression often correlates with increased tumor malignancy involving unstable 3p21.3 locus activity, the mechanism regulating HYAL1 expression and other genes at this locus in cancer cells remains poorly understood.

Transcriptional regulation of estrogen target genes is mediated through direct interaction with the estrogen receptors ERα (NR3A1) and ERβ (NR3A2), which belong to the nuclear hormone receptor family of ligand activated transcription factors [22]. ERα and ERβ bind to their cognate estrogen responsive element (ERE) in target promoters to mediate transcriptional regulation of estrogen-responsive genes. Interestingly, ERα-negative breast cancer cells, which tend to be more aggressive, exhibit enhanced hyaluronidase secretion when compared to ERα-positive cells [23]. We reported a similar inverse correlation for epithelial ovarian cancers in which clear cell and mucinous subtypes showed strong expression of HYAL1 but low levels of ERα [17]. In contrast, in serous and endometrioid tumors expressing high levels of ERα, HYAL1 was weakly expressed. In addition, ectopic expression of ERα in TOV21G ovarian cancer cells, which are derived from a clear cell carcinoma, resulted in a significant decrease in HYAL1 expression [17]. These findings support an inverse relationship between HYAL1 and ERα expression at least in ovarian and breast cancer cells, but the exact reason for such correlation remains undetermined.

In the current study, we show that the HYAL1 gene is a target of ERα in breast cancer cells. Analysis of the ERα cistrome identified several ERα binding sites within the 3p21.3 cluster, including in the vicinity of the HYAL1 gene, which were associated with histone marks in response to estrogen. Interestingly, among the 3p21.3 genes tested, only HYAL1 was found repressed by estrogen, indicating a selective role of these specific ERα binding elements to differentially support the estrogenic response of 3p21.3 genes in breast cancer cells.

## RESULTS

### HYAL1 expression is down-regulated by estrogen in breast cancer cells

**Figure 1:**
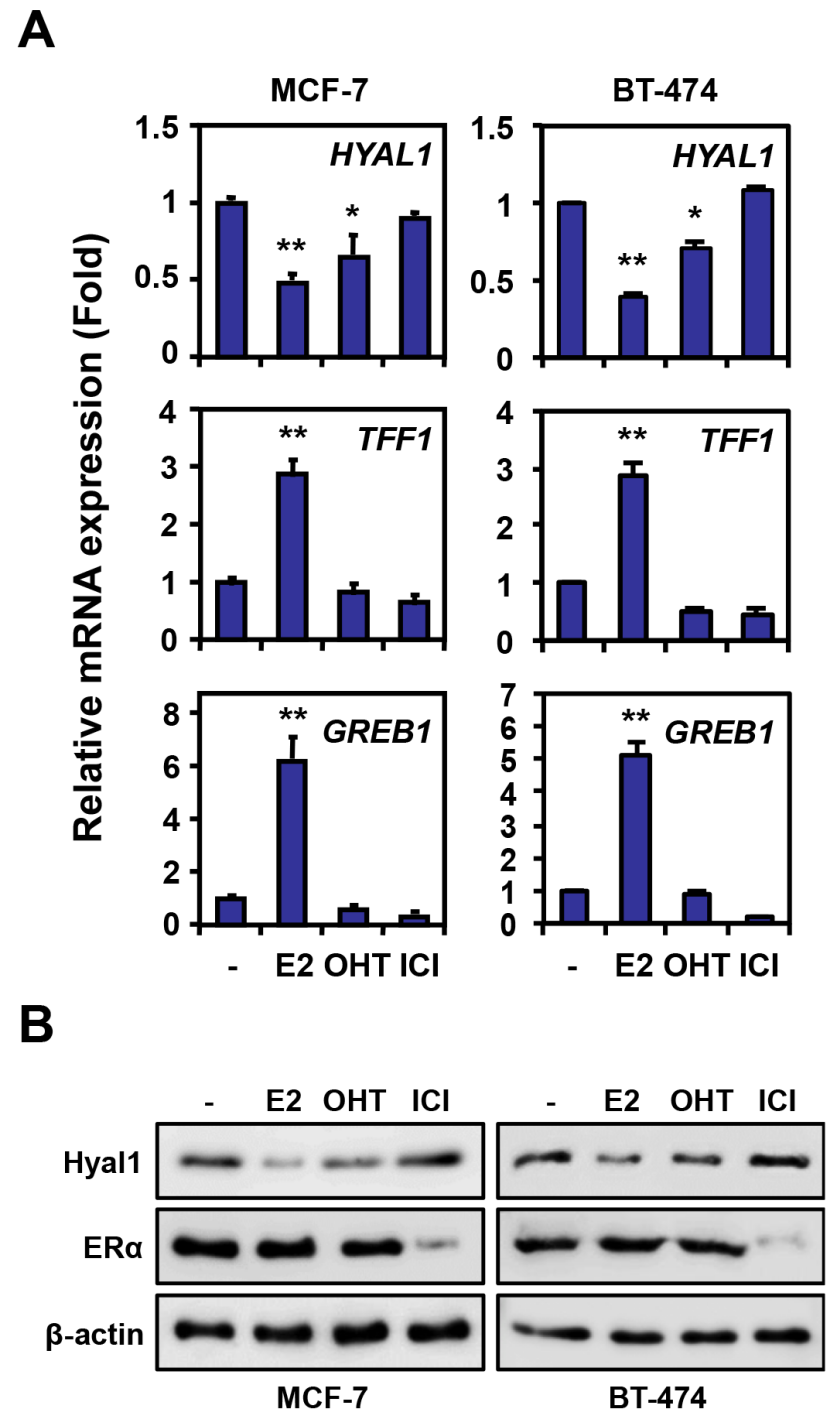
HYAL1 expression is downregulated by estrogen in breast cancer cells **A.** HYAL1 expression was measured by qPCR in MCF-7 and BT-474 breast cancer cells treated with vehicle (−), 10nM estradiol (E2), 100nM 4-hydroxytamoxifen (OHT) or 100nM ICI 182,780 for 16h. TFF1 and GREB1 are known estrogen-induced genes. Results were normalized with RPLP0 expression and expressed as fold response compared to vehicle-treated cells set at 1.0. *, P < 0.05; **, P < 0.005. **B.** Western analysis of HYAL1 and ERα in MCF-7 and BT-474 cells treated as in (A).

Our previous results have demonstrated an inverse correlation between HYAL1 and ERα expression in epithelial ovarian cancer cells, suggesting an estrogenic regulation of the HYAL1 gene in ERα positive cancer cells [17]. To investigate the possible regulation of HYAL1 by estrogen, we used human breast cancer MCF-7 cells, which are positive for ER and highly responsive to estrogen. When treated with 17β-estradiol (E2), MCF-7 cells exhibited a significant decrease in HYAL1 expression compared to control cells, whereas TFF1/pS2 and GREB1, two known estrogen inducible genes, were increased under the same conditions (Figure 1A, *left panel*). Such estrogenic decrease in HYAL1 expression was shown to take place in a time- and dose-dependent manner (Supplementary Figure S1). Similarly, HYAL1 was repressed in human breast cancer BT-474 cells, which also exhibit high expression of ERα (Figure 1A, *right panel*). Addition of the mixed antiestrogen tamoxifen also decreased HYAL1 expression in both cell lines, albeit to a lesser extent than with estrogen, whereas pure antiestrogen ICI 182,780, also known as fulvestrant, had no effect. Corresponding changes in protein levels were observed in MCF-7 and BT-474 cells with a stronger reduction of HYAL1 protein in response to estrogen, compared to tamoxifen and ICI 182,780 (Figure 1B). Under these conditions, ERα protein levels were moderately affected, except in presence of ICI 182,780, in accordance with its reported effect on ERα degradation [24]. Consistent with the inverse correlation of HYAL1 and estrogen, MCF-7 and BT-474 cells showed reduced basal levels of HYAL1 protein when compared to ERα-negative breast cancer MDA-MB-231 and ovarian epithelial cancer TOV21G cells, which are both strongly positive for HYAL1 (Supplementary Figure S2).

### Regulation of the 3p21.3 cluster genes by estrogen in breast cancer cells

**Figure 2:**
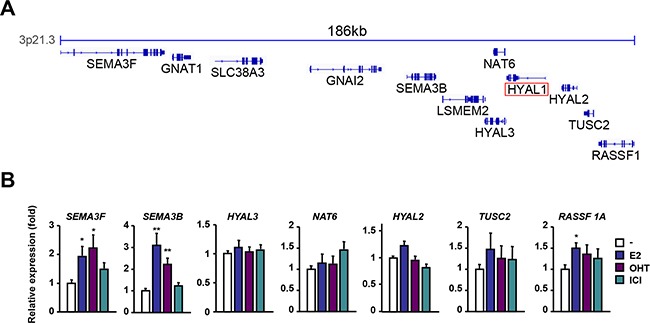
Regulation of the 3p21.3 locus by estrogen and antiestrogens **A.** Representation of the genes contained in the 3p21.3 locus using IGV. **B.** qPCR analysis of genes located in the vicinity of HYAL1 in the 3p21.3 locus. MCF-7 cells were treated with 10nM E2, 100nM OHT, or 100nM ICI 182,780 for 16h and results were normalized to RPLP0 expression and expressed as fold response compared to vehicle-treated cells set at 1.0. *, P < 0.05; **, P < 0.005.

We next addressed whether such down-regulation of HYAL1 was also occurring on the other closely located genes within the 3p21.3 locus as represented in Figure 2A. We found that the expression levels of SEMA3F, SEMA3B and RASSF1A genes were increased in MCF-7 cells in response to estrogen, whereas those of NAT6, TUSC2 and most notably HYAL2 and HYAL3 were not significantly changed compared to control cells (Figure 2B). This indicates that among the genes studied in the 3p21.3 locus, HYAL1 gene exhibits a selective response to estrogen, with a marked reduction in its expression. A similar expression profile was also observed in cells treated for a shorter period of time (*e.g.* 6 hrs), indicating a rapid response of 3p21.3 HYAL1, SEMA3B and SEMA3F genes to estrogen (Supplementary Figure S3). Treatment with tamoxifen or ICI 182,780 had no significant effect on the expression of most of the genes within the 3p21.3 cluster, except for SEMA3F and SEMA3B, which were increased by tamoxifen (Figure 2B).

### Selective down-regulation of HYAL1 by ERα

**Figure 3:**
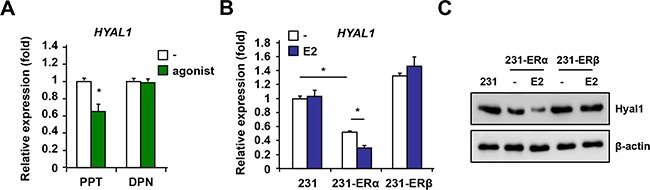
ERα but not ERβ mediates HYAL1 down-regulation in breast cancer cells **A.** MCF-7 cells were treated with selective agonist propylpyrazole-triol (PPT) for ERα or diarylpropionitrile (DPN) for ERβ for 16hrs and HYAL1 expression was analyzed by qPCR. Results were normalized to RPLP0 expression and are expressed as fold response compared to vehicle-treated cells set at 1.0. **B.** ERα and ERβ stable clones were generated from parental ER-negative MDA-MB-231 breast cancer cells and treated or not with 10nM estradiol (E2) for 16hrs. qPCR analysis was performed and results were normalized to RPLP0 expression and expressed as fold response compared to vehicle-treated cells set at 1.0. **C.** ERα selective decrease of HYAL1 protein levels. Western analysis in ER stably expressed 231 clones treated as in B. Control loading was normalized to β-actin. *, P < 0.05.

Because MCF-7 cells are positive for both ERα and ERβ isoforms, the respective contribution of each isoform in down-regulating HYAL1 with estrogen is difficult to ascertain. To address this, we treated MCF-7 cells with the selective agonist propylpyrazole-triol (PPT) for ERα or diarylpropionitrile (DPN) for ERβ and analyzed HYAL1 expression. We found that PPT significantly decreased HYAL1 levels in contrast to DPN which had no effect (Figure 3A), suggesting a predominant role of ERα to down-regulate HYAL1. To further define the respective role of ERα and ERβ in HYAL1 regulation, we generated stable clonal lines using ER-negative human breast cancer MDA-MB-231 cells, which are positive for HYAL1 (Supplementary Figure S2), and in which ERα (231-ERα) or ERβ (231-ERβ) was stably expressed and functional (Supplementary Figure S4). HYAL1 gene expression was found significantly decreased in 231-ERα cells treated with estrogen compared to untreated cells, whereas no changes were observed in 231-ERβ cells (Figure 3B). In addition, HYAL1 protein levels were lower in 231-ERα cells compared to 231-mock or to 231-ERβ cells, and were further reduced by the addition of estrogen (Figure 3C). These results suggest that HYAL1 down-regulation by estrogen is mediated through ERα and not ERβ in the context of breast cancer cells.

### Inverse correlation of HYAL1 and ESR1 in breast cancer clinical samples

**Figure 4:**
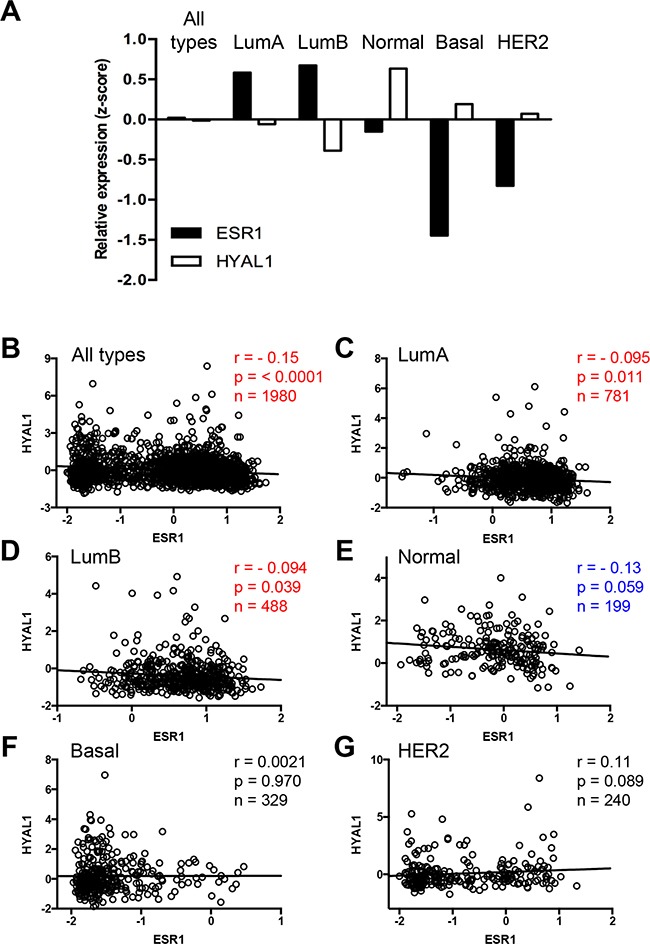
Inverse correlation of HYAL1 and ESR1 expression in breast cancer tissues **A.** Mean values of HYAL1 (white bars) and ESR1 (black bars) gene expression, from normalized z-scores of the METABRIC dataset, were plotted according to PAM50 breast cancer subtype stratification. **B-G.** Pearson correlation analysis was performed between HYAL1 and ESR1 gene expression (z-scores) in breast cancer tumors from the whole METABRIC cohort (n=1980) (B), or from luminal A (n=781) (C), luminal B (n=488) (D), normal-like (n=199) (E), basal-like (n=329) (F) and HER2-enriched (n=240) (G) breast tumor subtypes. In each case, Pearson scores and P values are indicated.

To determine the clinical relevance of our findings, we interrogated the METABRIC gene expression microarray dataset consisting of 1980 clinically annotated breast cancer specimens [25–27]. Integrative data mining analysis was performed to plot the mean values of mRNA expression for ESR1 (ERα gene) and HYAL1 in the whole cohort and in each of the breast cancer subtypes, i.e. luminal A, luminal B, normal-like, basal-like and HER2, as classified by the PAM50 assay [28, 29]. Our results show a striking inverse correlation between the expression levels of these two genes in all breast cancer subtypes (Figure 4A). In the whole cohort, values are close to zero because gene expression were normalized and expressed as z-score values (see Methods for details). We further conducted a more specific correlation study taking into account expression values in each patient. We thus performed Pearson correlation analysis which revealed a significant negative correlation (Pearson r = − 0.15, *p* < 0.0001, n=1980) between ESR1 and HYAL1 in the whole cohort, as well as in luminal A (Pearson r = − 0.095, *p* = 0.011, n=781) and luminal B (Pearson r = − 0.094, *p* = 0.039, n=488) subtypes (Figure 4B–4D, Table 1), which are the two subtypes with positive ESR1 expression (Figure 4A). An inverse correlation almost reaching significance was also observed in the normal-like subtype (Pearson r = − 0.13, *p* = 0.059, n=199) (Figure 4E), whereas no significant correlation was measured for basal-like and HER2-enriched subtypes (Figure 4F–4G). Furthermore, we found no significant correlation between gene expression of ESR1 and HYAL2, between ESR1 and HYAL3, or between ESR2 (ERβ gene) and HYAL1 (Table 1), consistent with our results in cells (Figures 2, 3, and Supplementary Figure S3). In addition, a significant positive correlation was observed between ESR1 and SEMA3B gene expression and ESR1 and SEMA3F (Table 1), two genes that were upregulated by estrogen (Figures 2 and Supplementary Figure S3). These findings indicate that HYAL1 regulation by ERα is specific and clinically relevant.

**Table 1:** Pearson correlation analysis applied to the METABRIC dataset (n=1980 breast cancer patients, whole cohort) for expression between estrogen receptor α (ESR1) and genes of the 3p21.3 cluster. Also shown is analysis between estrogen receptor β (ESR2) and HYAL1

Genes	Pearson r	p value
ESR1 *vs* HYAL1	−0.15	<0.0001
ESR1 *vs* HYAL2	0.0045	0.8427
ESR1 *vs* HYAL3	−0.0065	0.7739
ESR1 *vs* SEMA3B	0.45	<0.0001
ESR1 *vs* SEMA3F	0.48	<0.0001
ESR2 *vs* HYAL1	0.030	0.1847

### ERα cistrome analysis of the 3p21.3 cluster

**Figure 5:**
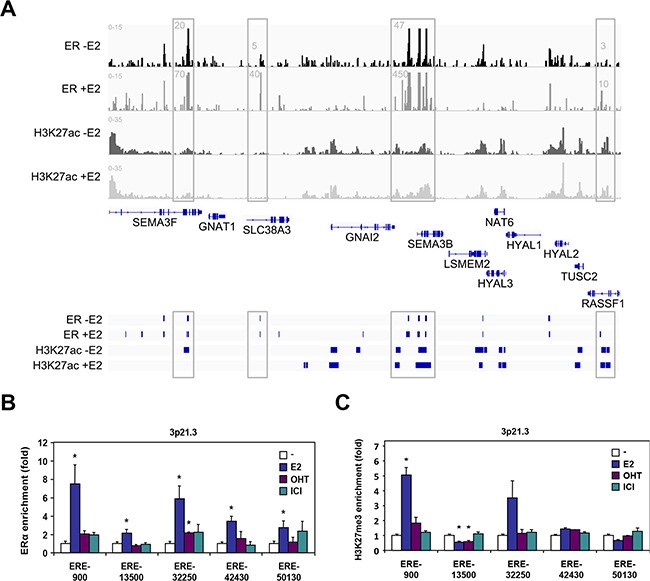
ERα binding analysis at the 3p21.3 locus **A.** UCSC genome view of ERα and H3K27ac enrichment at the 3p21.3 locus as determined by ChIP-seq analysis in MCF-7 cells. Peaks were mapped from cells treated with vehicle and 10nM estradiol for 45min, and aligned on the reference genome hg19. **B.** ChIP-qPCR validation of ERα binding sites identified from ChIP-seq analysis in vicinity of the HYAL1 gene. Positions of the estrogen response elements (ERE) are indicated relative to the transcriptional start site of the HYAL1 gene. MCF-7 cells were treated with 10nM E2, 100nM OHT, or 100nM ICI 182,780 for 45min. **C.** ChIP-qPCR mapping of repressive histone H3K27me3 mark relative to ERα binding sites analyzed in (B). Cells were treated as in (B).

Given the prominent role of ERα in HYAL1 down-regulation, we then performed ERα ChIP-seq data analysis in order to identify putative ERα binding sites in the vicinity of the HYAL1 gene and within the 3p21.3 cluster. Thus, we identified 69452 ERα binding sites in MCF-7 treated cells. Interestingly, our analysis revealed several binding sites occupied by ERα in the 3p21.3 cluster in absence and in presence of estrogen (Figure 5A). In particular, several of the estrogen-induced ERα binding sites were located in the vicinity of SEMA3B, SEMA3F, and RASSF1, three genes we found upregulated by E2 (Figure 2B and Supplementary Figure S3), suggesting that these sites are primarily used to confer estrogen activation. To delineate whether enrichment of the identified ERα binding sites by estrogen were associated with active transcription, we performed ChIP-Seq analysis under the same conditions to map the acetylated form of lysine 27 of histone 3 (H3K27Ac), a histone mark associated with transcriptional activation. As expected, ERα binding sites along the SEMA3B, SEMA3F and RASSF1 genes correlated with H3K27Ac mark, indicating the potential role of these sites in mediating estrogen-activation of these genes (Figure 5A). Similarly, enrichment of ERα and H3K27Ac mark was also found around the HYAL3 gene (HYAL3 is encoded by the bottom DNA strand), but this region does not seem to confer estrogen activation of HYAL3 since no significant regulation in expression was observed in treated MCF-7 cells (Figure 2B and Supplementary Figure S3) and ESR1 and HYAL3 gene expression did not correlate in breast tumors (Table 1). Interestingly, the genomic region (up to ~10kb) located 5′ to the HYAL1 gene (reverse orientation) did not display significant enrichment in H3K27Ac mark in presence of estrogen, suggesting a weakly responsive state of chromatin near HYAL1 in these conditions. However, our analysis has identified one ERα binding site located within the proximal promoter of HYAL1 (*i.e.* ERE-900), along with other distal sites, such as ERE-13500 and ERE-32250, located toward respectively HYAL2 and RASSF1.

**Figure 6:**
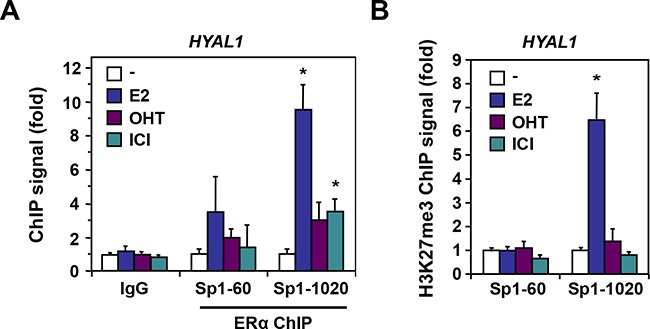
ERα enrichment at Sp1 binding sites of the HYAL1 promoter **A.** ChIP of ERα was performed at Sp1 binding sites located in the proximal promoter of HYAL1 in MCF-7 cells treated or not with 10nM E2, 100nM OHT, or 100nM ICI 182,780 for 45min. PCR was done with primers encompassing the proximal (−60) and distal (−1020) Sp1 binding sites relative to the transcriptional start site of the HYAL1 gene. **B.** Mapping of H3K27me3 repressive mark to the Sp1 sites of HYAL1. MCF-7 cells were treated as in (A).

We thus performed ChIP-qPCR analysis to validate these ERα binding sites identified within the 3p21.3 cluster near the HYAL1 gene. Control ChIP experiments showed a strong recruitment of ERα at the TFF1 gene (Supplementary Figure S5). Under the same conditions, we found that all the binding sites tested within the 3p21.3 locus identified from our ChIP-Seq analysis were able to recruit ERα in MCF-7 cells treated with estrogen, whereas the antiestrogens did not allow significant enrichment (Figure 5B). Of interest, the ERE-900, ERE-32250, and to a lesser extent ERE-42430 were found to be the most enriched ERα binding sites in response to estrogen, when compared to other EREs of the cluster (*e.g.* ERE-13500 and ERE-50130). To address whether these sites correlate with transcriptional repression in accordance with the down regulation of HYAL1 by estrogen (Figure 1A and Figure 3), we performed ChIP assay for the histone H3K27me3 repressive mark. The methylation of H3K27 is catalyzed by the polycomb repressive complex 2 (PRC2) member enhancer of zeste homolog 2 (EZH2/KMT6A), which has been associated with repression of estrogen-responsive genes and with the severity and progression of breast cancer [30, 31]. We found that the ERE-900 was strongly associated with the H3K27me3 mark in presence of estrogen, in contrast to more distal sites (Figure 5C), suggesting that the ERE-900 located in the proximal promoter of HYAL1 is linked to estrogenic repression. Sequence analysis near the ERE-900 revealed the presence of two putative Sp1 binding sites at positions -60 and -1020. Besides direct interaction with EREs, estrogen regulation of ERα target genes can be mediated through direct interactions with other transcription factors, such as Sp1 [32]. We found a significant enrichment of ERα to the Sp1-1020 site compared to the Sp1-60 in response to estrogen (Figure 6A). This enrichment was accompanied by a strong increase in H3K27me3 mark at the Sp1-1020 site (Figure 6B). These results suggest that the repressive action of estrogen on HYAL1 implies ERα recruitment to the ERE-900, probably depending on the adjacent Sp1 binding region, and that such recruitment might cooperate with the PRC2 complex to induce H3K27me3 enrichment at the HYAL1 promoter.

### Estrogen repression of the HYAL1 promoter requires ERE-900

**Figure 7:**
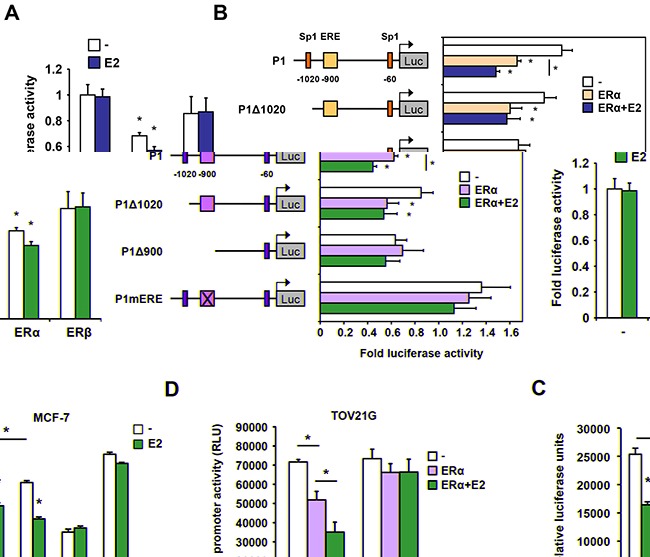
Regulation of the proximal promoter activity of HYAL1 by ERα **A.** 293 cells were transfected with ERα or ERβ in the presence of a luciferase reporter construct containing the proximal P1 promoter of HYAL1. Cells were then treated or not with 10nM estradiol (E2) for 16h. Luciferase values were normalized to β-galactosidase activity and expressed as relative fold response compared to vehicle-treated mock transfected cells set at 1.0. **B.** Truncated regions of the HYAL1 proximal P1 promoter were analyzed for estrogen responsiveness in luciferase reporter assays as in (A) in response to ERα expression. The ERE-900 was also mutated (mERE) in the context of the P1 fragment. Cells were treated or not with 10nM estradiol (E2) for 16h. Luciferase values were normalized to β-galactosidase activity and expressed as relative fold response compared to vehicle-treated controls set at 1.0. **C.** HYAL1 wild-type, truncated, or mutated P1 promoter activity was determined in MCF-7 cells treated with 10nM E2 or vehicle for 16h. Cells were analyzed for luciferase activity as in (A). **D.** Same as in (C) except that ovarian cancer TOV21G cells were used. *, P < 0.05.

We isolated a 1282 bp fragment of the proximal region immediately flanking the transcription start site of HYAL1 and tested its response to estrogen using luciferase assay. We found that in 293 cells transfected with ERα, the HYAL1 promoter region, containing the ERE-900 and the Sp1-1020 sites, conferred transcriptional repression in response to estrogen (Figure 7A). In contrast to ERα, ERβ expression was inefficient in promoting a similar repression, in accordance with results obtained using selective agonists and stable 231 cell lines (Figure 3). 5′ deletions of the HYAL1 promoter showed that removal of the Sp1-1020 impaired the repressive action of estrogen and further removal of the ERE-900 completely abolished both the effect of ERα ectopic expression and estrogen treatment, compared to full-length promoter (Figure 7B). A disrupting site mutation of the ERE-900 also abolished these effects, suggesting an indispensable role of the ERE-900 in mediating HYAL1 promoter repression. Consistent with this, the ERE-900 was found essential for the repressive action of estrogen in MCF-7 (Figure 7C) and in BT-474 (Supplementary Figure S6) breast cancer cells, while deletion of the Sp1-1020 site slightly diminished but did not abolish the down-regulation by estrogen. Likewise, disruption of the ERE-900 completely impaired the repressive effect of ERα expression and activation on HYAL1 promoter in TOV21G ovarian epithelial cancer cells (Figure 7D), in which HYAL1 expression is known to inversely correlate with ERα (Supplementary Figure S2 and ref. [17]). These results suggest a predominant role of ERE-900 in ERα-mediated repression of HYAL1 in estrogen responsive cancer cells.

## DISCUSSION

Increasing evidence supports a role of hyaluronidase family members in tumor progression and metastasis, but the exact regulatory mechanisms underlying the highly variable and context-dependent expression profiles of HYAL genes in terms of cancer type and severity still remain poorly understood. Here, we report a distinct and selective response to estrogen among the 3p21.3 locus genes in breast cancer cells, where the HYAL1 gene was repressed as compared to HYAL2 and HYAL3, and to other genes tested within the cluster. This negative regulation of HYAL1 by ERα provides a mechanism supporting the specific inverse correlation we found between ESR1 and HYAL1, but not with HYAL2 or HYAL3 in the METABRIC cohort consisting of 1980 cases of breast cancer. Despite the fact that most of ERα binding sites present in the 3p21.3 locus were associated with active chromatin marks in response to estrogen, HYAL1 expression was decreased by estrogen treatment, accompanied by the enrichment of repressive H3K27me3 mark. These results identify HYAL1 as an ERα target gene with clinical relevance and suggest a differential role of the specific ERα binding elements in the selective regulation of the 3p21.3 genes in response to estrogen.

Considerable work has been made to improve our understanding of hormone-dependent gene activation by ERα, but relatively little is known about how estrogen represses gene expression. Our results demonstrate that estrogen repression of HYAL1 involves direct binding of ERα to the ERE-900 element located in the proximal HYAL1 promoter region, accompanied with enrichment of the repressive H3K27 tri-methylation mark. Aberrant epigenetic events, including histone modifications, are central to alter gene expression profiles, a hallmark of cancer development. In breast cancer, histone hypermethylated marks are frequently associated to genes involved in cell-cycle regulation, apoptosis, tissue invasion and metastasis [33, 34]. In particular, the methyltransferase EZH2/KMT6A and its end-product H3K27me3 mark are found elevated in aggressive breast cancer subtypes, including triple negative and ERα-positive tumors resistant to endocrine therapies, suggesting inactivation of tumor suppressor genes [31, 35–38]. Our results demonstrate that H3K27 methylation profile associated with the ERα binding sites identified throughout the 3p21.3 cluster is highly variable, suggesting the potential of estrogen to selectively up- or down-regulate specific genes within this cluster. Accordingly, some of these ERE elements were associated with the transcriptionally active H3K27 acetylation mark, probably sustaining a positive response to estrogen for genes such as SEMA3B, SEMA3F and RASSF1A. Class 3 semaphorins B and F are candidate tumor suppressors and their increased response to estrogen has been reported in ovarian cancer cells, resulting in inhibition of cell growth [39–41]. Whether these effects are translated through the same EREs identified in this study is not known but our results indicate that a similar estrogen up-regulation of SEMA3B and 3F expression is also taking place in breast cancer cells, and is consistent with the positive correlation we found between ESR1 and SEMA3B or SEMA3F in human breast tumors using METABRIC dataset.

For HYAL1 repressive response, the exact mechanism as to how estrogen can induce H3K27 tri-methylation at the HYAL1 promoter is not clearly known. However, based on recent evidence showing that EZH2 can be transcriptionally induced by estrogen in breast cancer cells [42], this might provide a mechanism by which the methylation of H3K27 can be promoted by estrogen in conjunction with the direct recruitment of ERα at the HYAL1 ERE-900. Consistent with this, ERα recruitment and H3K27me3 repressive mark enrichment at HYAL1 promoter is strongly correlated since treatment with antiestrogen ICI-182,780 or tamoxifen did not result in significant changes in ERα and H3K27me3 levels at the ERE-900. Such requirement of specific epigenetic landmarks was found essential to provide stringent response of ERα to estrogen [43], as exemplified for the estrogen-responsive BCL2 promoter [44], thereby supporting a role of the chromatin environment to impose ligand dependency of ERα activity. Interestingly, estrogen was shown to promote interaction of ERα but not ERβ with EZH2 on target promoters of treated MCF-7 cells [45]. Such ER isoform selective effect is consistent with our findings on the ability of ERα and not ERβ to repress the HYAL1 gene. Whether the enrichment of the H3K27me3 mark at the HYAL1 promoter requires prior ERα binding and/or specific EZH2 recruitment to estrogen-bound ERα at the ERE-900 element is not known, but the repressive chromatin landscape triggered by estrogen in the region of HYAL1 appears highly specific when compared to other distal EREs located in the 3p21.3 cluster. Indeed, most of the validated EREs shown to recruit ERα in response to estrogen were not enriched with H3K27me3 repressive mark, suggesting that estrogen promotes a gene-specific chromatin remodelling at the 3p21.3 cluster.

We examined the involvement of Sp1 binding sites in translating estrogen responsiveness of HYAL1 and found a contributing role of Sp1-1020 site located in the vicinity of ERE-900. A functional Sp1 binding element has been correlated with low HYAL1 expression in bladder and prostate cancer cells [46], suggesting that Sp1 might impair the promoter activity of HYAL1. However, this Sp1 element, which corresponds to Sp1-60 in this study, did not seem to promote the repressive action of estrogen in breast cancer cells. Sp1 is an interaction partner of ERα, which in most cases confers estrogen responsiveness to its target genes without the prior requirement of ERα binding to an ERE [32]. In the HYAL1 response to estrogen, both the ERE-900 and the Sp1-1020 elements seem to be required, suggesting that prior binding of ERα and Sp1 to their respective sites is needed to achieve optimal HYAL1 repression. This appears not to be a common aspect of estrogen-responsive genes that are regulated via Sp1-ERα interaction, which in most cases do not require direct ERα binding to DNA [32, 47–49]. For HYAL1, both the ERE and Sp1 sites are required to confer optimal estrogenic downregulation, adding to the possible actions of ERα-mediated repression. Whole genome location analyses have depicted enrichment of Sp1 binding sites in close proximity of EREs, which better predict functional ERα/Sp1 complex interaction and response to estrogen [50–52].

In addition, our observation of enhanced estrogenic ERα recruitment around the HYAL1 promoter is not always a shared feature for repressed genes. For example, estrogenic down-regulation of BTG2 and E-Cadherin genes does not involve increased ERα occupancy at their respective promoters [48, 53]. Such induced recruitment of ERα at the HYAL1 promoter might therefore promote appropriate changes in the chromatin environment leading to histone modifications such as the H3K27me3 mark as suggested by our data. Additional changes might as well implicate recruitment of transcriptional corepressors. For example, nuclear receptor corepressors NCoR and SMRT have been shown to be recruited at promoters of ERα/Sp1 repressed genes, such as VEFGR2 and CCNG2 [47, 54]. However, we did not find any changes in NCoR/SMRT recruitment at the HYAL1 promoter in response to estrogen (data not shown), suggesting a possible involvement of other cofactors yet to be identified. For instance, coregulators such as RIP140 and REA have been associated to the repressive actions of estrogen on respectively PROS1 and BTG2 genes in the context of ERα and Sp1 [48, 55]. Whether these or other coregulators are involved in ERα-mediated HYAL1 repression remains to be investigated. However, our results outline a possible mechanism that mediates estrogen-dependent down-regulation of HYAL1, which contributes to expand the diverse nature of processes used by ERα to regulate gene repression.

Chromosomal instability at the 3p21.3 locus and aberrant expression of hyaluronidase family members associated to deleterious effects of hyaluronan breakdown end-products have been linked with poor prognosis and metastasis of many epithelial cancers [13–15]. However, expression levels of HYAL1/2/3 isoforms were found to be highly variable depending of cancer types, raising the complexity of regulatory mechanisms involved. Our identification of ERα as a regulatory transcriptional component of the 3p21.3 genes, and in particular of HYAL1, reveals a probable link between breast cancer subtypes and metastatic potential. Upregulation of HYAL1 has been observed in infiltrating invasive duct tumors and metastatic lymph nodes and shown to promote migration potential [19–21]. Our data mining analysis using the METABRIC dataset did reveal a significant negative correlation between HYAL1 and ESR1 genes in breast tumors, providing a clinical relevance to the direct down-regulation of HYAL1 by estrogen. We have also reported such negative relationship in ovarian tumors, in which poor prognosis morphological subtypes, such as clear cell and mucinous tumors, exhibit high HYAL1 expression levels that are inversely correlated with ERα expression [17]. Such stratification based on HYAL1 expression in concordance with ERα status in breast cancer subtypes is suggested with our results showing significant lower expression of HYAL1 in breast tumors highly expressing ESR1, such as luminal A and luminal B subtypes. Furthermore, we observed a reduction of HYAL1 expression in response to estrogen in ERα-positive breast cancer cells and to ectopic expression of ERα in ERα-negative cells. Interestingly, these effects solely relied on ERα and not ERβ, which has a limited clinical value [56, 57] and did not reach significance when compared to HYAL1 expression in this study. In such context, it would be interesting to address the potential of HYAL1 upregulation to associate with metastatic phenotype and/or tumor resistance resulting in poor prognosis. Consistent with such HYAL1 increase, low molecular weight products of hyaluronan breakdown were enhanced in aggressive breast tumors and ERα-negative cell lines, providing these fragments with a potential clinical value for breast cancer metastasis [58].

In conclusion, the regulation of gene expression at the 3p21.3 locus is certainly complex and diverse with a direct impact on tumorigenesis in cases of aberrant regulation. Our study provides a functional framework for the direct action of ERα and hormonal regulation of 3p21.3 genes and on its repressive effect on HYAL1 gene with clinical relevance. Understanding the mechanism of HYAL1 gene repression as part of the physiopathological actions of estrogen should provide essential insights and reveal potential therapeutic strategies.

## MATERIALS AND METHODS

### Cell culture and treatments

Human breast cancer MCF-7 and BT-474 cells were routinely maintained in DMEM supplemented with 10% FBS (Wisent Inc., St-Bruno, QC). ER-negative human MDA-MB-231 breast cancer cells were transfected with expression vectors for ERα and ERβ and resistant clones were selected with neomycin to generate 231-ERα and 231-ERβ cell lines, respectively. Stable clones were functionally validated for their respective expression of ERα and ERβ by Western analysis and for their estrogenic response by luciferase assay. Human epithelial ovarian cancer TOV21G cells were derived from clear cell carcinoma [17] and cultured in OSE medium supplemented with 10% FBS (Wisent Inc.). Human embryonic kidney 293 cells were maintained in DMEM and 5% FBS. Unless otherwise stated, cells were treated with 10nM 17β-estradiol (E2; Sigma), 100nM 4-hydroxy-tamoxifen (OHT) and 100nM ICI 182,780 and compared to vehicle-treated cells. Cells were also treated with specific agonist propylpyrazole-triol (PPT) for ERα or diarylpropionitrile (DPN) for ERβ obtained from Tocris.

### HYAL1 promoter constructs and mutagenesis

The HYAL1 proximal promoter fragments were amplified by PCR according to GenBank sequence of the HYAL1 gene contig (NG_009295) and the UCSC hg19 genome assembly (https://genome.ucsc.edu). Amplified fragments corresponding to 1282pb (P1), 1060bp (P1Δ1020), and 746pb (P1Δ900) from HYAL1 transcriptional start site were inserted in front of the luciferase coding region in pBLuc plasmid as described [59]. Site-directed mutagenesis of the ERE at position -900 was performed by PCR. All constructs were validated using automated sequencing. The list of primers used appears in Supplementary Table S1.

### Transfection and luciferase reporter assay

Prior to transfection, cells were seeded in phenol red–free DMEM medium supplemented with 5% charcoal dextran–treated serum. MCF-7 and 293 cells were transfected by calcium phosphate precipitation as described [60], whereas TOV21G cells were transfected using GeneJuice transfection reagent according to the manufacturer's instruction (EMD Chemicals, Gibbstown, NJ). Typically, cells were transfected with 250ng of luciferase reporter, 10-50ng of pCMX-hERα or pCMX-hERβ, and 100ng β-gal construct per well and then treated with vehicle or 10 nM E2 for 16h. Cells are then harvested in potassium phosphate buffer containing 1% Triton X-100 and lysates analyzed for luciferase activity using a plate reader (Perkin-Elmer). Luciferase values are normalized for transfection efficiency to β-galactosidase activity and expressed as relative fold response compared with controls. Luciferase assays are performed in duplicates from at least three independent experiments.

### RNA extraction and qPCR analysis

Total RNA was isolated using a commercial kit (Promega, Madison, WI) according to the manufacturer's instruction and cDNA was prepared using the iScript cDNA Synthesis kit (Bio-Rad, Mississauga, ON). Quantitative PCR amplification was performed as described [61] using a MX3000P cycler (Agilent, Santa-Clara, CA). Relative expression values were normalized to RPLP0 or β-actin and compared to control samples. Each analysis was performed in duplicates and results are derived from at least three independent experiments. Primer sequences are listed in Supplementary Table S1.

### Chromatin immunoprecipitation (ChIP) assay

MCF-7 cells were grown to 70-80% confluence and serum-deprived in phenol red-free DMEM/F-12 medium before treatment with vehicle (control), 10nM E2, 100nM 4-OHT or 100nM ICI 182,780 for 45 min. Chromatin was harvested and ChIP and qPCR was performed as described [59, 62]. Primer sequences are listed in Supplementary Table S1. Each analysis was performed in duplicates and results are derived from at least four independent ChIP experiments.

### Western analysis

Western analysis was carried out in MCF-7 and BT-474 cells, and in ERα- and ERβ-231 stable clones treated as above and compared to vehicle (control) treatment. Cells were then harvested as described [59] and immunoblotting was performed using antibodies to HYAL1 (GeneTex, Irvine, CA) and ERα (Santa Cruz Biotech, Santa Cruz, CA). In each experiment, total protein loading was normalized using an anti-β-actin antibody (Novus Biologicals, Oakville, ON).

### ChIP-seq analysis

The ChIP-seq data were generated as follow: after trimming [63], the sequenced reads were aligned against the human reference genome hg19 using BWA [64], then peaks were called by MACS [65] using default parameters. Aligned tags were converted to Bedgraph files by HOMER [66] and UCSC, and visualized with IGV [67]. ERα ChIP-seq data were downloaded from [68], while H3K27ac ChIP-seq was generated as described [69].

### Cell lysates and immunoblotting analysis

Immunoblotting procedures were done essentially as described [59, 62]. Briefly, cells were washed in ice-cold PBS and lysed in Tris-buffered saline (TBS) containing 0.1% Triton X-100, 1 mM orthovanadate, 1 mM NaF, 0.1 mM PMSF, and protease inhibitors (Roche, Laval, QC). Lysate samples (20-30 μg protein) were then resolved by SDS-PAGE and transferred onto nitrocellulose. Membranes were blocked with blocking reagent (Roche) or with 5% dehydrated skim milk in TBS, probed with selected antibodies at 4°C, and incubated with appropriate horseradish peroxidase conjugated secondary antibodies. Signals were captured by enhanced chemiluminescence (Perkin Elmer, Boston, MA) and analyzed using a LAS 4000 imaging system (GE Life Science, Mississauga, ON).

### METABRIC gene expression data

Gene microarray expression (Affymetrix U133) for HYAL1, HYAL2, HYAL3, ESR1, ESR2, SEMA3B and SEMA3F were directly downloaded from the cBioportal for Cancer Genomics website (www.cbioportal.org) using the METABRIC breast cancer dataset [25–27]. Gene expression is normalized to the distribution of each gene in tumors and is annotated as z-scores. Breast cancer subtypes of each tumor sample, classified by the PAM50 assay [28, 29], were also downloaded from the cBioportal website. Graph Pad Prism 6 was used to perform Pearson correlation test (two-tailed) and significance was set at P < 0.05.

## SUPPLEMENTARY MATERIALS FIGURES AND TABLE




