# Supplementary material for: Proximal and distal regulation of the HYAL1 gene cluster by the estrogen receptor α in breast cancer cells
Source: Oncotarget. 2016 Oct 13;7(47):77276–90. doi: 10.18632/oncotarget.12630 (PMC5363586; doi:10.18632/oncotarget.12630)
Supplement: Supplementary file 1 [file oncotarget-07-77276-s001.pdf]

Proximal and distal regulation of the HYAL1 gene cluster by the estrogen receptor α in breast cancer cells

SUPPLEMENTARY FIGURES AND TABLE

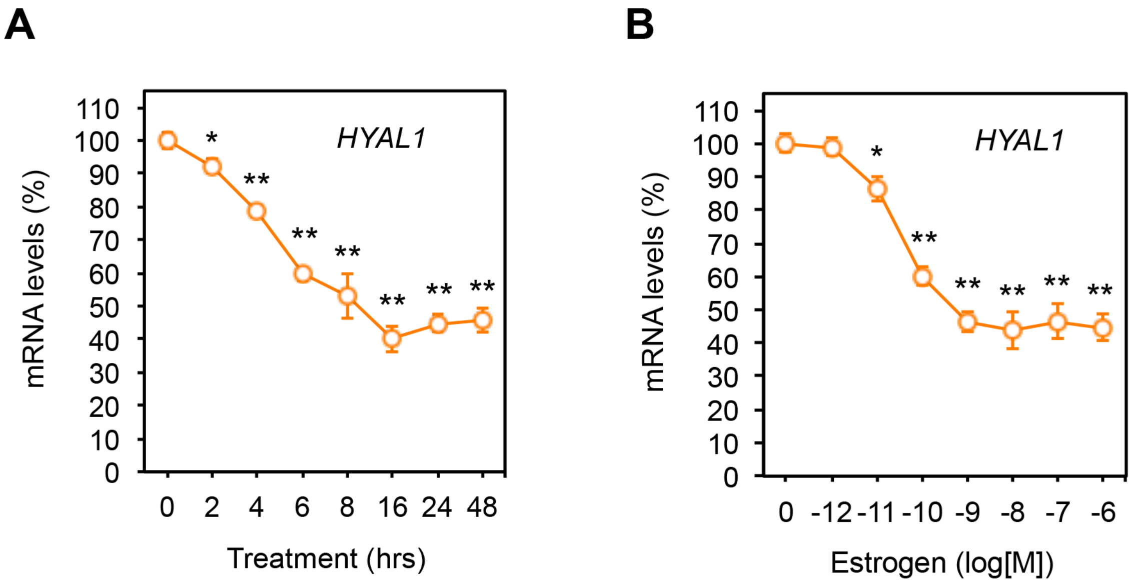

Supplementary Figure S1: Estrogen regulation of HYAL1 gene in breat cancer cells. A. Time-dependent decrease of HYAL1 expression by estrogen. Real-time PCR analysis of HYAL1 expression in human breast cancer MCF-7 cells treated with 10^{-8}M 17β-estradiol for the indicated time periods. Results were normalized to RPLP0 expression and expressed as % response compared to vehicle-treated cells set at 100. Values represent means ± SDs derived from three separate experiments performed in triplicates. **B.** Similar as in A, except that cells were treated with increasing concentrations of 17β-estradiol for 16hrs. *, P<0.02; **, P<0.001 *vs* control cells.

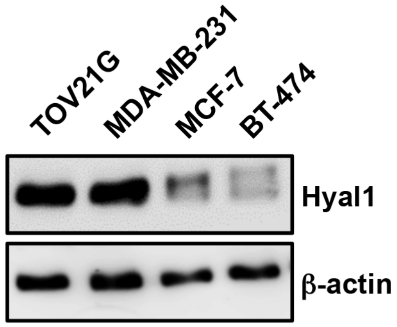

Supplementary Figure S2: Relative Hyal1 protein expression in breast and ovarian cancer cells. Western blot analysis of Hyal1 in ERα-positive breast cancer cells (MCF-7 and BT-474) and ERα-negative MDA-MB-231 breast cancer and TOV21G ovarian cancer cells. Total protein loading was normalized using an anti-β-actin antibody.

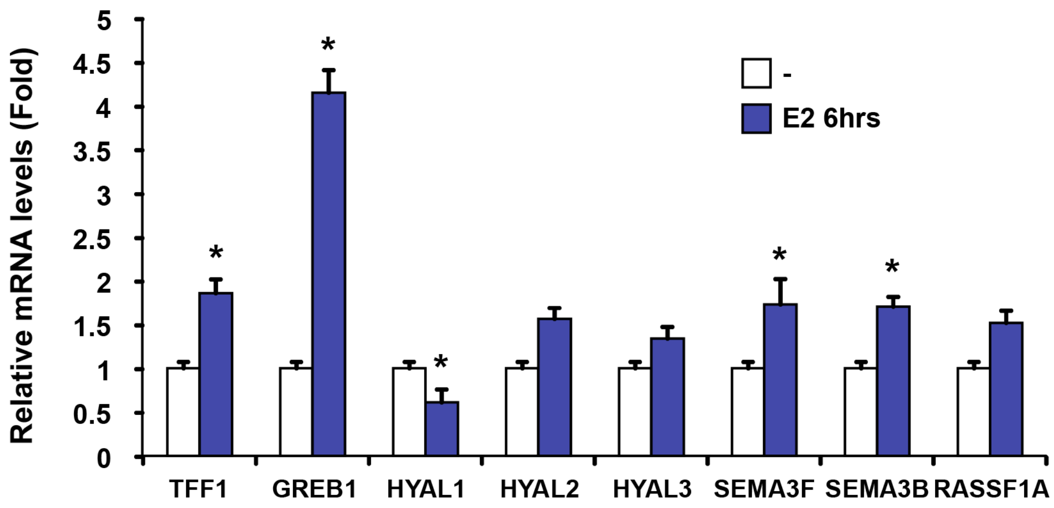

Supplementary Figure S3: Regulation of the 3p21.3 locus by estrogen. Real-time PCR analysis of genes located in the 3p21.3 locus. MCF-7 cells were treated with 10nM E2 for 6hrs and results were normalized to RPLP0 expression and expressed as fold response compared to vehicle-treated cells set at 1.0. Also represented are estrogen-responsive genes TFF1 and GREB1. *, P < 0.05.

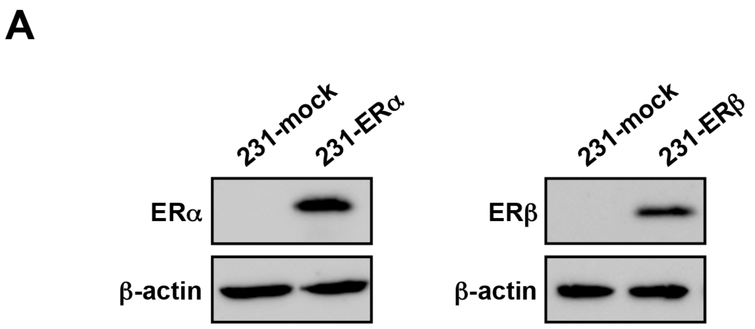

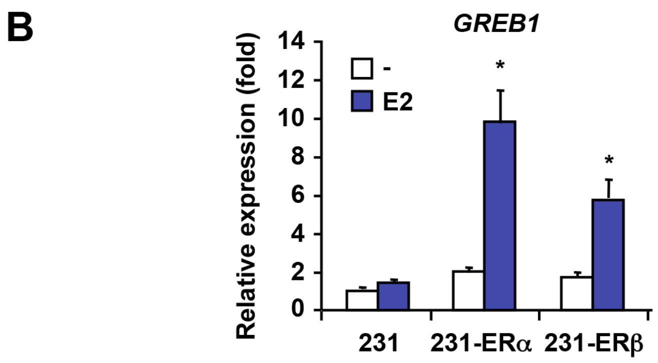

Supplementary Figure S4: MDA-MB-231 cell lines stably expressing ERα and ERβ. ER-negative human MDA-MB-231 breast cancer cells were transfected with expression vectors for ERα and ERβ and resistant clones were selected with neomycin to generate 231-ERα and 231-ERβ cell lines, respectively. **A.** Cells were analyzed for respective ERα (*left*) and ERβ (*right*) expression by Western blot and compared with mock-transfected control cells. **B.** ER target GREB1 gene expression was analyzed by qPCR in 231 stable clones. Cells were treated or not with 10nM estradiol (E2) for 16hrs. Results were normalized to RPLP0 expression and expressed as fold response compared to vehicle-treated 231 control cells set at 1.0. *, P < 0.05.

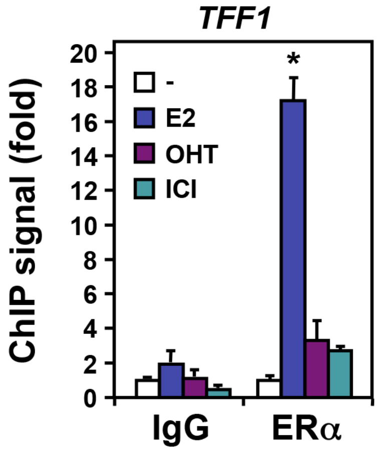

Supplementary Figure S5: Induction of ERα enrichment to TFF1 gene by estrogen. Validation of ChIP-qPCR analysis of the ER target gene TFF1 in MCF-7 cells treated with 10nM estradiol, 100nM tamoxifen and 100nM ICI 182,780 for 45min. Results are expressed as fold-enrichment of ERα binding compared to vehicle-treated cells set at 1.0. IgG was used as a negative control. *, $P < 0.05$.

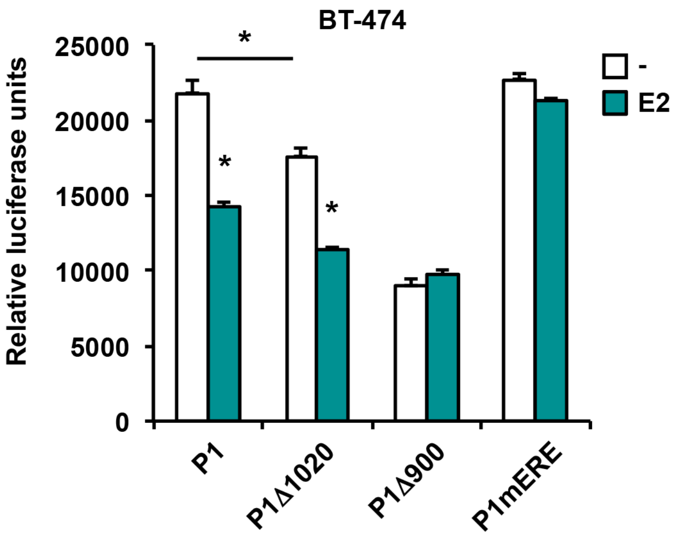

Supplementary Figure S6: Estrogenic repression of the proximal promoter of HYAL1 in breast cancer cells. ERα-positive breast cancer BT-474 cells were transfected with luciferase reporter gene under the control of the proximal promoter (P1) of HYAL1 or truncated variants (Δ900 and Δ 1020). The ERE-900 was also mutated (mERE) in the context of the P1 fragment. Cells were then treated or not with 10nM estradiol (E2) for 16h and harvested for luciferase assay. Luciferase values were normalized to β-galactosidase activity and are expressed as relative luciferase units. *, $P < 0.05$ *vs* vehicle-treated cells for each construct.

Supplementary Table S1: List of primers used.

See Supplementary File 1
