# Supplementary material for: Proximal and distal regulation of the HYAL1 gene cluster by the estrogen receptor α in breast cancer cells
Source: Oncotarget. 2016 Oct 13;7(47):77276–90. doi: 10.18632/oncotarget.12630 (PMC5363586; doi:10.18632/oncotarget.12630)
Supplement: Supplementary file 2 [file oncotarget-07-77276-s002.docx]

**Table S1:** List of primers used

| **qRT-PCR analysis** | |
| --- | --- |
| **HYAL1** | forward 5'-AAGCCCTCCTCCTCCTTAACC-3'  reverse 5'-AGCCAGGGTAGCATCGAC-3' |
| **HYAL2** | forward 5'-CACCACAAGCACGGAGACCT-3'  reverse 5'-CAGGCACTAGGCGGAAACTG-3' |
| **HYAL3** | forward 5'-TGCTGGCATCTCCATGACTACC-3'  reverse 5'-CTTCCATCTGTCCTGGATCTCG-3' |
| **SEMA3F** | forward 5'-CGTGAGGAACCCTGTCATTTACGC-3'  reverse 5'-CCGTGGGTAGGGCATCTTCCCTG-3' |
| **SEMA3B** | forward 5'-CCAGCGCAGCCTGGTCAACAAGTG-3'  reverse 5'-GGACGTGGAGAAGACGGCATAGAG-3' |
| **RASSF1A** | forward 5'-CCGTGGCCACCGCTTCCAGCCC-3'  reverse 5'-CCGCAACAGTCCAGGCAGACGAG-3' |
| **TUSC2** | forward 5'-TCACCAAGAACGGGCAGAAG-3'  reverse 5'-TCATAGAGGATCACAGGGAAATCC-3' |
| **NAT6** | forward 5'-ACTTGAAGCAGCACCCGTTG-3'  reverse 5'-ACCACCACTGTCTCCACTAAGAGG-3' |
| **TFF1** | forward 5'-TGGAGAACAAGGTGATCTGCG-3'  reverse 5'-CGAAACAGCAGCCCTTATTTGC-3' |
| **GREB1** | forward 5'-TTCCCCGAAGTGCCAACAAC-3'  reverse 5'-ATGGAGATTCTGGAGACCACCC-3' |

**Table S1:** List of primers used (cont’d)

| **ChIP-qPCR analysis** | |
| --- | --- |
| **ERE-900** | forward 5'-TAACCCAGACATGACCTCCTGG-3'  reverse 5'-GGCAATGTAGCAAGACCCCATC-3' |
| **ERE-13500** | forward 5'-CCCTGAGGAAAGAAGGAGCAG-3'  reverse 5'-GGTGAGTCATCACGGACATGG-3' |
| **ERE-32250** | forward 5'-CGGTGGCTCACACCTCTAATG-3'  reverse 5'-GCTGACCTCGAATTCCTGATTG-3' |
| **ERE-42430** | forward 5'-ATAAGGAGGAGGAAGAGCAGCG-3'  reverse 5'-TGCCAATCGCCATAGTATTTGG-3' |
| **ERE-50130** | forward 5'-ACATGGAGGGGCAAATCCAG-3'  reverse 5'-TGTGCTCACTCTGGTTCACTGC-3' |
| **Sp1-60** | forward 5'-AACCAAGATCCCTTTGCCAG-3'  reverse 5'-TCCAAATTTCCTGACCCCAG-3' |
| **Sp1-1020** | forward 5'-CACTAAGCACAATAGACCCAAGGCC-3'  reverse 5'-GAGGTCATGTCTGGGTTACACTATT-3' |
| **HYAL1 promoter** | |
| **P1-F** | 5’-GACACAGTCACTAACCCAGC-3’ |
| **P1-Δ900-F** | 5’-AGCACAGTGGAGCAGTCTTG-3’ |
| **P1-Δ1020-F** | 5’-GTGTAACCCAGACATGACC-3’ |
| **P1-R** | 5’-TCCAAATTTCCTGACCCCAG-3’ |
| **MutERE-F** | 5’-GACTACAGGTACATAAAACCAAACCCAGCTAATTTT-3’ |
